# Moving Object Detection Based on Fusion of Depth Information and RGB Features

**DOI:** 10.3390/s22134702

**Published:** 2022-06-22

**Authors:** Xin Bi, Shichao Yang, Panpan Tong

**Affiliations:** School of Automotive Studies, Tongji University, Shanghai 201804, China; bixin@tongji.edu.cn (X.B.); yangshichao@tongji.edu.cn (S.Y.)

**Keywords:** moving object detection, RGB-D, convolutional neural network CNN, super-resolution reconstruction

## Abstract

The detection of moving objects is one of the key problems in the field of computer vision. It is very important to detect moving objects accurately and rapidly for automatic driving. In this paper, we propose an improved moving object detection method to overcome the disadvantages of the RGB information-only-based method in detecting moving objects that are susceptible to shadow interference and illumination changes by adding depth information. Firstly, a convolutional neural network (CNN) based on the color edge-guided super-resolution reconstruction of depth maps is proposed to perform super-resolution reconstruction of low-resolution depth images obtained by depth cameras. Secondly, the RGB-D moving object detection algorithm is based on fusing the depth information of the same scene with RGB features for detection. Finally, in order to evaluate the effectiveness of the algorithm proposed in this paper, the Middlebury 2005 dataset and the SBM-RGBD dataset are successively used for testing. The experimental results show that our super-resolution reconstruction algorithm achieves the best results among the six commonly used algorithms, and our moving object detection algorithm improves the detection accuracy by up to 18.2%, 9.87% and 40.2% in three scenes, respectively, compared with the original algorithm, and it achieves the best results compared with the other three recent RGB-D-based methods. The algorithm proposed in this paper can better overcome the interference caused by shadow or illumination changes and detect moving objects more accurately.

## 1. Introduction

In recent years, with the rapid development of computer vision and machine learning technologies, moving target detection based on video sequence images has become an important research topic in the field of computer vision [1]. Moving target detection refers to the segmentation of moving objects and stationary regions in an image sequence or video, and the objects with spatial position changes are extracted as the foreground [2].It involves computer vision, machine learning, video processing and other fields. The accurate and rapid detection of moving objects is of great significance to intelligent driving, intelligent video monitoring, medical treatment and even military aspects. For example, in the traffic system, moving object detection technology is used to obtain the current traffic flow on the road; in the military field, moving target detection can be used to achieve accurate guidance.

Traditional moving object detection methods are based only on the information of RGB images taken by 2D cameras to extract objects, which can reflect the information of object color and light intensity to a certain extent, but it cannot comprehensively consider the object’s spatial location and three-dimensional information such as size and shape. In addition, the use of color images to detect objects is susceptible to the effects of shadows, occlusion and illumination, mainly for the following reasons: the target and its shadow mostly have the same motion properties, and both have large differences from the background, so it is easy to misjudge the shadow as the target; occlusion will lead to the loss of tracking targets; illumination changes will lead to significant changes in the image, which is not conducive to the separation of background and foreground.

This paper focuses on the above problem and proposes an innovative moving target detection algorithm that fuses depth and RGB information based on the codebook algorithm [3]. The depth information of the same scene is used to compensate for the inaccuracy of recognition due to shadow interference, light change or color interference when only color images are used for detection. The two main aspects are super-resolution reconstruction of depth images and fusion of depth and color information for the detection of moving targets. Our contribution can be summarized as follows:

- In this paper, we propose a color edge-guided depth map super-resolution CNN, which uses the correspondence between the color map and the depth map to input both the low-resolution depth map and the high-resolution color-optimized edge map of the same scene into the network, and it uses the edge map to guide the depth map reconstruction. The final experimental results show that the proposed model effectively improves the quality of the depth map after super-resolution.
- We propose an RGB-D based codebook moving object detection algorithm, which adds the depth information of the scene as an auxiliary dimension based on the original codebook algorithm, including two parts of background modeling and foreground detection, and it takes advantage of the depth information not being disturbed by shadows, illumination and occlusion to better segment moving objects.

The rest of the paper is organized as follows. Section 2 describes existing studies related to super-resolution image reconstruction and moving object detection for depth images. Section 3 presents our super-resolution reconstruction model and moving object detection algorithm. Section 4 shows the comparison of our method with existing methods. Finally, Section 5 concludes the paper and outlines future research directions.

## 2. Related Works

### 2.1. Depth Image Super-Resolution Reconstruction

In terms of super-resolution reconstruction using only a single image, Freeman et al. [4] formulated the super-resolution problem into a multi-class Markov Random Field (MRF) model, where each hidden node represents the label of a high-resolution block. However, the reconstruction process is highly dependent on the available training samples, and it may not provide reliable results when correspondence cannot be established. Dong et al. [5] integrated the traditional sparsely coded image super-resolution method process into a CNN to learn the mapping between low and high-resolution images directly during the training process, which provides higher accuracy while achieving simplification and speediness purposes. However, this method does not utilize any prior knowledge, and it is sometimes difficult to learn such many-to-one mappings, resulting in problems such as blurring or artifacts. Based on the example depth map, Mandal et al. [6] constructed the sub-dictionary of the example to restore the high-resolution depth map. Lei et al. [7] proposed a trilateral depth mapping up-sampling method based on viewpoint synthesis quality, which considered the depth smoothness, texture similarity and viewpoint synthesis quality in the up-sampling filter.

With the development of deep learning, super-resolution based on deep convolutional networks is a rapidly growing field [8], and several classes of super-resolution CNNs have emerged. The linear Super-Resolution Convolutional Neural Network (SRCNN) [5],which was first used for super-resolution, is regarded as a pioneering work in super-resolution based on deep learning and has had a great impact in the field of super-resolution. Later, the deeper network Very Deep Super-Resolution (VDSR) [9] was proposed, and the accuracy after reconstruction was further improved as the network deepened. In addition to these, there are the super-resolution residual network Cascading Residual Network (CARN) [10], recursive network Super-Resolution Feedback Network (SRFBN) [11], and the attention-based network Holistic Attention Network (HAN) [12]; the success of these models demonstrates the usability of convolutional neural networks in the super-resolution domain.

In terms of reconstruction using multiple depth images, Rajagopalan et al. [13] modeled the original high-resolution image into MRF according to the Bayesian framework, and they used the edge adaptive MRF to deal with discontinuity, which achieved good results. Wetzl et al. [14] used the pseudo-Huber loss function as the regularization term of the objective function, and they used the quasi-Newton optimizer to calculate the minimization. With the help of Graphics Processing Unit (GPU) acceleration, high-resolution images can be restored at an interactive frame rate. Izadi et al. [15] only use depth data from Kinect to track the 3D attitude of the sensor and reconstruct the geometric accurate 3D model of the physical scene in real time.

From the aspect of color image-guided depth map super-resolution reconstruction, Ferstl et al. [16] developed a second-order TGV model based on the assumption that RGB images correspond to depth image edges, using the raw RGB intensity as input to the anisotropic diffusion tensor to enhance image detail as well as edge retention, and solving it numerically using a primal-pairing algorithm. Zhang [17] introduced the wavelet transform-extracted edge indicator function into the color-constrained canonical term on this basis to make the details clearer at the edges as well as in the images. Yang et al. [18] proposed an adaptive color-guided autoregressive model, which is constructed by the local correlation in the initial depth map and the nonlocal similarity in the color image. Song [19] proposed a progressive deep CNN framework and further optimized the reconstructed depth images by combining the constraint of having local consistency between color and depth images, and the constraint of statistical information of depth images. Wen et al. [20] proposed a coarse-to-fine CNN and constructed a color guidance strategy for depth image up-sampling based on color difference and spatial distance.

### 2.2. Moving Target Detection

In terms of a moving object detection algorithm, the traditional methods mainly include the inter-frame difference method [21,22], optical flow method [23,24] and background difference method [25,26]. The inter-frame difference method is generally used to extract moving objects from adjacent frames in video, and the background difference method [27] is used to detect the target by performing the difference operation between the current video frame and the background model. In recent years, with the progress of depth image acquisition technology, there are many moving target detection algorithms based on RGB-D information. On the basis of a 2D moving target detection algorithm, the depth information of the object is added to eliminate the interference of shadow, illumination change and occlusion. Parvizi et al. [28] defined the splitter by analyzing the local minimum of the probability density function for depth information, reducing over-segmentation or under-segmentation to some extent. Ottonelli et al. [29] used the ViBE (visual background extractor) background modeling algorithm to segment human contours with high reliability, and they added the compensation factor obtained by color and depth information, but it still has the problems of edge noise and missing detection. Hu et al. [30] established a Bayesian classifier at each pixel based on color and depth information, and then, they fused it to detect moving objects. Hu et al. [31] improved the reliability of a color map and depth map with different weights under three different illumination conditions by integrating the feature information of color space and depth space in the background model updating stage of the ViBE algorithm. Li et al. [32] proposed a moving object detection method combining a sequential frame difference method and optical flow algorithm with higher accuracy and efficiency. They first divided the pre-processed images in pairs of sequences and calculated the sum of the differential results, and then, they processed the differential video sequences using the optical flow method to accurately detect and identify moving targets. However, it has a large amount of calculation.

It can be seen that detection based on RGB information alone is susceptible to illumination changes and shadow interference, resulting in low detection accuracy and noise, while some other algorithms have high detection accuracy but are computationally intensive. On the other hand, it also shows that depth information has a strong application value in the field of moving object detection. Therefore, this paper first studies the super-resolution reconstruction of a depth map, proposes a CNN model framework for the super-resolution reconstruction of a depth map based on color edge guidance, and obtains a high-quality depth map. Then, the codebook target detection algorithm is improved by using depth information. A moving object detection algorithm based on depth information and RGB features fusion is proposed, which can show excellent performance in scenes such as illumination change and shadow interference, and it provides a reference for target detection methods in complex scenes.

## 3. Proposed Method

### 3.1. Depth Map Super-Resolution Reconstruction Based on Color Edge Guidance

Image super-resolution reconstruction technology has become one of the hotspots of image research in recent years. Due to the imperfect manufacturing process of a depth camera, it is difficult to obtain high-resolution depth maps. In addition, the traditional deep image super-resolution technology often has the defects of blurred image edges and increased noise after reconstruction. With the development of deep learning technology, researchers have gradually applied deep learning technology to the field of image super-resolution reconstruction and achieved good effects. Inspired by the SRCNN proposed by Dong [5] and the dual-flow network described by Liu [33], and considering the guiding effect of the edge information of high-resolution color images on the super-resolution reconstruction of a depth map, this section proposes a convolution neural network framework with dual-input sources. The depth image and the optimized edge map extracted from the color image of the same scene are input into the network at the same time. The color information is used to assist the depth map to complete the reconstruction and improve the quality of the reconstructed image.

To facilitate the description below, the meaning of the variable symbols used in this section is explained as follows. *I* represents the high-resolution color map, $D_{l}$ represents the low-resolution depth map, $D_{H}$ represents the high-resolution depth true value map, $I_{bw}$ represents the optimized edge map, and $D_{L}$ represents the low-resolution depth map of the same size as *I*.

#### 3.1.1. Edge Graph Preprocessing

**Figure 1:**
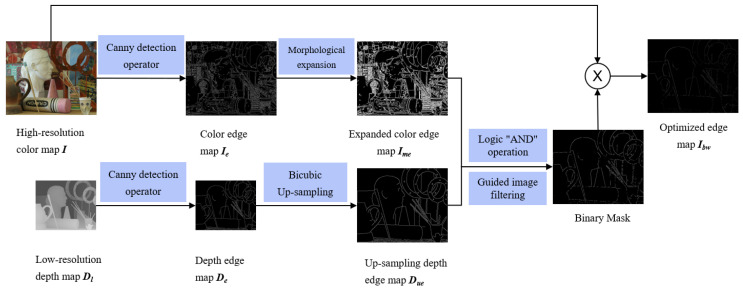
Process of Obtaining the Optimized Color Edge Map $I_{bw}$.

The proposed method needs to be preprocessed before inputting the image into the CNN, including obtaining the low-resolution depth map $D_{L}$ with the same size as the color map *I* and the edge map $I_{bw}$ of color and depth fusion. $D_{L}$ is obtained by using the bicubic up-sampling method for $D_{l}$, and the method for obtaining $I_{bw}$ refers to Reference [34]. Firstly, canny edge detection operator is performed on $D_{l}$ and *I* to obtain the depth edge map $D_{e}$ and color edge map $I_{e}$, respectively. Then, the bicubic interpolation method is used to sample the depth edge map, and the up-sampling depth edge map $D_{ue}$ with the same size as the color edge map is obtained. In addition, in order to prevent the misalignment of the RGB image and the depth image, the morphological expansion operation is performed on the color edge map $I_{e}$ to obtain the expanded color edge map $I_{me}$, and then, the logical “AND” operation is performed between the expanded color edge map $I_{me}$ and the up-sampled depth edge map $D_{ue}$. Finally, to prevent noise at the edges, the edge map is smoothed using a Guided Image Filter [35], which effectively preserves the image edges, and $I_{bw}$ is obtained. The process of obtaining the optimized color edge map $I_{bw}$ is shown in Figure 1.

#### 3.1.2. Network Model Framework

**Figure 2:**
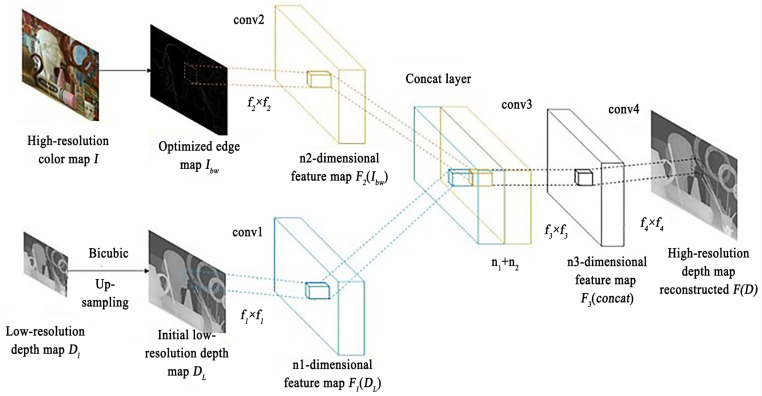
CNN for Super-Resolution Reconstruction.

The CNN framework designed in this paper is shown in Figure 2. The input low-resolution depth map $D_{L}$ and the optimized color edge map $I_{bw}$ are firstly extracted from the features of the two maps through the upper and lower convolutional layers conv1 and conv2, respectively. The operations of these two convolutional layers can be expressed as follows:

(1) $$F_{1}\left(D_{L}\right)=max\left(0,W_{1}⊗D_{L}+b_{1}\right)+a_{1}min\left(0,W_{1}⊗D_{L}+b_{1}\right)$$

(2) $$F_{2}\left(I_{bw}\right)=max\left(0,W_{2}⊗I_{bw}+b_{2}\right)+a_{2}min\left(0,W_{2}⊗D_{L}+b_{2}\right)$$

Here, $W_{1}$, $W_{2}$ and $b_{1}$, $b_{2}$ represent the weight and bias coefficient, respectively, symbol ⊗ represents convolution, $W_{1}$ corresponds to a number of $n_{1}$ convolution kernels of size $c\times f_{1}\times f_{1}$, where *c* represents the number of channels of the input image, and $f_{1}\times f_{1}$ is the size of the convolution kernels. The number of output feature maps is $n_{1}$, and $b_{1}$ is the $n_{1}$-dimensional vector. $W_{2}$ and $b_{2}$ are similar to the above. Finally, PRelu is used as the activation function, while $a_{1}$ and $a_{2}$ are $n_{1}$-dimensional and $n_{2}$-dimensional vectors, respectively, which are adaptive coefficients in PRelu.

The concat layer splices $F_{1}\left(D_{L}\right)$ and $F_{2}\left(I_{bw}\right)$ with the channel as the axis to integrate the depth feature and color feature. This step can be expressed as:

(3) $$concat=F_{1}\left(D_{L}\right)⊕F_{2}\left(I_{bw}\right)$$

where the symbol ⊕ indicates the stitching with the channel as the axis.

The third convolution layer realizes the mapping from a low-resolution map to a high-resolution map. Finally, the fourth convolution layer is reconstructed to obtain a high-resolution depth map. These two steps can be expressed as:

(4) $$F_{3}\left(concat\right)=max\left(0,W_{3}⊗concat+b_{3}\right)+a_{3}min\left(0,W_{3}⊗concat+b_{3}\right)$$

(5) $$F\left(D\right)=W_{4}⊗F_{3}\left(concat\right)+b_{4}$$

where $W_{3}$ corresponds to a number of $n_{3}$ convolution kernels of size $\left(n_{1}+n_{2}\right)\times f_{3}\times f_{3}$, $W_{4}$ corresponds to a number of *c* convolution kernels of size $n_{3}\times f_{4}\times f_{4}$, and $b_{3}$ and $b_{4}$ are $n_{1}$-dimensional and $n_{2}$-dimensional vectors, respectively.

### 3.2. Codebook Moving Target Detection Algorithm Based on RGB-D

#### 3.2.1. Algorithm Overview

**Figure 3:**
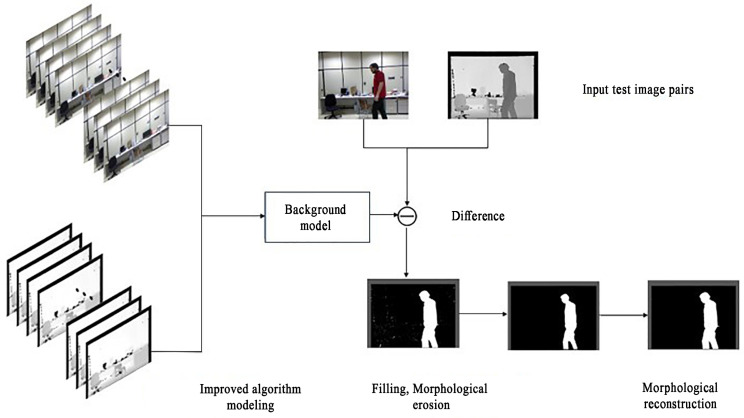
The improved RGB-D based codebook moving object detection algorithm framework in this paper.

Traditional moving object detection algorithms typically use RGB color information to detect the moving objects in the video, but its detection effect is poor due to the influence of shadow, occlusion and illumination mutation. In addition, there are also problems such as a large number of dense noise points or blurred edges in the detection results. On the contrary, the depth information is mostly unaffected by shadows and illuminations. When the depth information is added to the background modeling, the target detection algorithm with more accurate detection results and better robustness can be theoretically obtained. Therefore, the moving object detection algorithm proposed in this paper is based on the codebook algorithm and adds the depth information as an extension. The color and depth information of the pixel are combined to comprehensively judge whether the pixel belongs to the foreground or background. Finally, redundant noise points are removed, and the detection results are optimized by filling holes, morphological erosion and morphological reconstruction. The algorithm is implemented by MATLAB, and the algorithm framework is shown in Figure 3.

#### 3.2.2. Specific Algorithm Flow

The codebook algorithm proposed by Kim et al. firstly inputs the video frame sequence; then, it uses the idea of clustering to classify each pixel, adds codewords one by one, and updates the codebook regularly to obtain the final background model. It occupies small memory, and the intuitive principle of the method is simple, including background modeling and foreground detection. The following will briefly introduce them, respectively:

- Background modeling of the original codebook algorithm

The codebook algorithm firstly uses the input *N* video frames for training. $\chi =\{x_{1},x_{2},...,x_{N}\}$ is used to represent that each pixel has an *N*-dimensional RGB vector, and $l=\{c_{1},c_{2},...,c_{L}\}$ is used to represent that each pixel has a codebook with *L* codewords. The codebook model of each pixel contains different numbers of codewords according to the change of the sample. Each codeword $c_{i}$ consists of an RGB vector $v_{i}$ and a cell ${aux}_{i}$ with six elements, where:

(6) $$\left\{\begin{array}{c} v_{i}=\left({\bar{R}}_{i},{\bar{G}}_{i},{\bar{B}}_{i}\right)\\ {aux}_{i}=<{\overset{ˇ}{I}}_{i},{\hat{I}}_{i},f_{i},\lambda_{i},p_{i},q_{i}> \end{array}\right.$$

where ${\bar{R}}_{i},{\bar{G}}_{i},$ and ${\bar{B}}_{i}$, respectively, represent the average value of the color components of the pixels belonging to the codeword $c_{i}$, ${\overset{ˇ}{I}}_{i}$ and ${\hat{I}}_{i}$ represent the minimum and maximum light intensity values of the pixels belonging to the code $c_{i}$, respectively, $f_{i}$ represents the frequency of occurrence of the codeword $c_{i}$, $\lambda_{i}$ represents the maximum interval in which the code word $c_{i}$ is not reproduced during the training phase, and $p_{i}$ and $q_{i}$ represent the first and last time of the codeword $c_{i}$, respectively.

In the training phase, each pixel is compared with all the codewords in the current codebook to determine whether they can be matched to the codewords. The specific content of the algorithm is shown in Algorithm 1:
**Algorithm 1** Codebook background modeling algorithm**Input:** 
N three-channel video frames, $\varepsilon_{1}$, α, β, $T_{M}$**Output:** 
a background model1:Initialize L=0, set l to empty set2:**for**t=1 to *N* **do**3:$x_{t}=\left(R,G,B\right),I=\sqrt{R^{2}+G^{2}+B^{2}}$4:   **for** each $c_{m}\in l$ **do**5:     **if** colordist($x_{t}$,$v_{m}$)≦$\varepsilon_{1}$ brightness(*I*,<${\overset{ˇ}{I}}_{m}$, ${\hat{I}}_{m}$ >)=true **then**6:        $v_{m}=\left(\frac{f_{m}{\bar{R}}_{m}+R}{f_{m}+1},\frac{f_{m}{\bar{G}}_{m}+G}{f_{m}+1},\frac{f_{m}{\bar{B}}_{m}+B}{f_{m}+1}\right)$7:        ${aux}_{m}=<min\left\{I,{\overset{ˇ}{I}}_{m}\right\},max\left\{I,{\hat{I}}_{m}\right\},f_{m}+1,max\left\{\lambda_{m},t-q_{m}\right\},p_{m},t>$8:     **else**9:        L=L+110:        create a new $c_{L}$ by setting11:        $v_{L}=\left(R,G,B\right)$12:        ${aux}_{L}=<I,I,1,t-1,t,t>$13:     **end if**14:   **end for**15:**end for**16:**for** each i=1 to *L* **do**17:   $\lambda_{i}=max\left\{\lambda_{i},\left(N-q_{i}+p_{i}-1\right)\right\}$18:**end for**

$\varepsilon_{1}$ in the algorithm is a manually given threshold, and the two judgment conditions represent the color distortion and brightness limits, respectively. For the input pixel $x_{t}$ and codeword $c_{i}$, there are:

(7) $$\left\{\begin{array}{c} \left|\right|x_{t}{\left|\right|}^{2}=R^{2}+G^{2}+B^{2},\\ \left|\right|v_{i}{\left|\right|}^{2}={\bar{R}}_{i}^{2}+{\bar{G}}_{i}^{2}+{\bar{B}}_{i}^{2},\\ <x_{t},v_{i}{>}^{2}={\left({\bar{R}}_{i}R+{\bar{G}}_{i}G+{\bar{B}}_{i}B\right)}^{2} \end{array}\right.$$

The color distortion can be calculated as:

(8) $$\left\{\begin{array}{c} p^{2}=\left|\right|x_{t}{\left|\right|}^{2}cos^{2}\theta =\frac{<x_{t},v_{i}{>}^{2}}{\left|\right|v_{i}{\left|\right|}^{2}},\\ colordist\left(x_{t},v_{i}\right)=\sqrt{\left|\right|x_{t}{\left|\right|}^{2}-p^{2}} \end{array}\right.$$

In order to allow the change of brightness during detection, the upper and lower limits of brightness have been given:

(9) $$\left\{\begin{array}{c} I_{low}=\alpha \hat{I},\\ I_{h}=min\left\{\beta \hat{I},\frac{\overset{ˇ}{I}}{\alpha }\right\} \end{array}\right.$$

where α is usually between 0.4 and 0.7, and β is usually between 1.1 and 1.5. Then:

(10) $$brightness\left(I,<\overset{ˇ}{I},\hat{I}>\right)=\left\{\begin{array}{cc} true, & if\;I_{low}≦\left|\right|x_{t}\left|\right|≦I_{h},\\ false, & otherwise \end{array}\right.$$

In the background update stage, according to the maximum interval time to remove the code words that have not appeared for a long time, the improved background model *M* is obtained:

(11) $$M=\{c_{m}|c_{m}\in l\cap \lambda_{m}≦T_{M}\}$$

where $T_{M}$ is the threshold, set to half the number of training frames, that is N/2.

- Foreground detection of original codebook algorithm

When the background model in the video scene is obtained, the codebook foreground detection algorithm can perform background subtraction BGS(x) operation on the new pixel input in the test set, which is fast and intuitive, as shown in Algorithm 2.
**Algorithm 2** Codebook foreground detection algorithm**Input:** 
pixel containing (*R, G, B*) components, $\varepsilon_{t}$, *M***Output:** 
type of pixel1:$x=\left(R,G,B\right),I=\sqrt{R^{2}+G^{2}+B^{2}}$2: **for** each $c_{m}\in M$ **do**3:   **if** $colordist\left(x_{t},v_{m}\right)≦\varepsilon_{t}$$brightness(I,<{\overset{ˇ}{I}}_{m},{\hat{I}}_{m}>)=true$ **then**4:     $v_{m}=\left(\frac{f_{m}{\bar{R}}_{m}+R}{f_{m}+1},\frac{f_{m}{\bar{G}}_{m}+G}{f_{m}+1},\frac{f_{m}{\bar{B}}_{m}+B}{f_{m}+1}\right)$5:     ${aux}_{m}=<min\left\{I,{\overset{ˇ}{I}}_{m}\right\},max\left\{I,{\hat{I}}_{m}\right\},f_{m}+1,max\left\{\lambda_{m},t-q_{m}\right\},p_{m},t>$6:     **return** false(background)7:   **else**8:     **return** true(foreground)9:   **end if**10:**end for**

$\varepsilon_{t}$ is the threshold for detection; if the pixel is not matched to the code word in the background model, the pixel is defined as foreground; otherwise, it is background.

**Figure 4:**
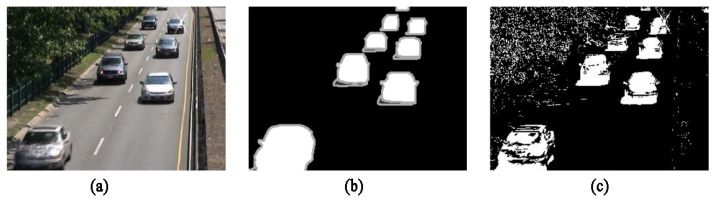
Comparison of the detection results of the original codebook algorithm: (**a**) Color image; (**b**) True value diagram; (**c**) Detection result diagram of the original codebook algorithm.

Although the detection speed of the original codebook algorithm is fast, color information is taken into account, and the upper and lower limits of light intensity are set to reduce the interference caused by light changes, the experimental results show that the effect is average. As shown in Figure 4, the highway video frame sequence in the baseline category in the CDNet2014 dataset [36] is used to evaluate the target detection effect of the original codebook algorithm.

It can be seen from Figure 4 that the original codebook algorithm can obtain the detection results quickly and can basically detect the approximate contour of the moving object. However, due to factors such as the leaves on the roadside swaying with the wind and changes in light intensity in the video, the original algorithm shows a large number of false detections at the trees beside the road on the upper left, and there are many missed detections inside the car. In addition, the shadows of the cars have the same trajectory as the cars and lead to a certain degree of adhesion between the shadows and the cars in the detection results, and more noise points appear elsewhere.

Considering that the shooting of the depth image is independent of brightness and color, this paper adds depth information to the codeword to expand the dimension of the codeword, and it optimizes the output result graph to reduce noise. Therefore, this paper proposes a codebook algorithm based on RGB-D to improve the performance and robustness of the algorithm. The algorithm includes background modeling and foreground detection.

- Codebook moving target detection algorithm based on RGB-D

The algorithm proposed in this paper adds one-dimensional depth information into the RGB component $v_{i}$ of the original codeword to convert into an RGBD vector, namely $v_{i}=\left({\bar{R}}_{i},{\bar{G}}_{i},{\bar{B}}_{i},{\bar{D}}_{i}\right)$. Then, we add the minimum and maximum depth values ${\overset{ˇ}{D}}_{i}$ and ${\hat{D}}_{i}$ of all the pixels belonging to codeword $c_{i}$ to the original six-dimensional tuple to make ${aux}_{i}$ an eight-dimensional tuple, namely ${aux}_{i}=<{\overset{ˇ}{I}}_{i},{\hat{I}}_{i},{\overset{ˇ}{D}}_{i},{\hat{D}}_{i},f_{i},\lambda_{i},p_{i},q_{i}>$. In addition, based on the two judgment conditions of background modeling and foreground detection algorithm, the depth deviation limit is added, and its definition refers to the light intensity limit, that is:

(12) $$depth\left(D,<\overset{ˇ}{D},\hat{D}>\right)=\left\{\begin{array}{cc} true, & if\;D_{low}≦D≦D_{h}\cup D=0,\\ false, & otherwise \end{array}\right.$$

where

(13) $$\left\{\begin{array}{c} D_{low}=\alpha_{d}\hat{D},\\ D_{h}=min\left\{\beta_{d}\hat{D},\frac{\hat{D}}{\alpha_{d}}\right\} \end{array}\right.$$

where $\alpha_{d}$ is usually between 0.4 and 0.7, $\beta_{d}$ is usually between 1.1 and 1.5. If the boundary range is too large, some foreground points will not be detected because the depth value changes little; if the range is too small, due to the quality problem of the depth map of the data set, the noise points whose depth values change are mistakenly detected as moving objects. Adding D=0 to the formula takes into account that the depth of the moving object may be empty. In the judgment, the algorithm proposed in this paper not only takes the intersection after comparing color and depth changes separately but also fuses the two in order to reduce the interference of shadow and light intensity changes with color changes as the dominant one. The judgment conditions are set as $color(x_{t},v_{i})$ and $bright(I,D,<\overset{ˇ}{I},\hat{I},\overset{ˇ}{D},\hat{D}>)$ as follows:

(14) $$color\left(x_{t},v_{i}\right)=\left\{\begin{array}{cc} true, & if\;colordist\left(x_{t},v_{i}\right)≦\varepsilon_{1}\;\cup \\ \; & \left(\varepsilon_{1}<colordist\left(x_{t},v_{i}\right)≦\varepsilon_{2}\cap depth\left(D,<\overset{ˇ}{D},\hat{D}>\right)\right)\\ false, & otherwise \end{array}\right.$$

(15) $$bright(I,D,<\overset{ˇ}{I},\hat{I},\overset{ˇ}{D},\hat{D}>=\left\{\begin{array}{cc} true, & if\;brightness(I,<\overset{ˇ}{I},\hat{I}>)\;\cup \\ \; & \left(brightness2\left(I,<\overset{ˇ}{I},\hat{I}>\right)\cap depth\left(D,<\overset{ˇ}{D},\hat{D}>\right)\right)\\ false, & otherwise \end{array}\right.$$

where $\varepsilon_{2}=k\varepsilon_{1},k>1$ is another threshold value greater than $\varepsilon_{1}$, $brightness2(I,<\overset{ˇ}{I},\hat{I}>$ is the value when $brightness(I,<\overset{ˇ}{I},\hat{I}>$ takes another set of $\alpha_{2}$ and $\beta_{2}$, making the upper and lower limits larger. The movement of shadow and the change of illumination will inevitably change the color and light intensity of the corresponding pixel, but the depth value of the pixel is not affected. Therefore, these two equations show whether the change of depth value exceeds the threshold as an auxiliary. Although the color and brightness of the pixel change, the depth change is still within the set range, which largely excludes the possibility that the pixel is a moving object.

${\bar{D}}_{i}$, ${\overset{ˇ}{D}}_{i}$, and${\hat{D}}_{i}$ is updated in the same way as ${\bar{R}}_{i}$, ${\overset{ˇ}{I}}_{i}$, and${\hat{I}}_{i}$, where three conditions are required to determine whether a pixel $x_{t}$ has a matching code $c_{m}$, namely:

(16) $$\left\{\begin{array}{c} color(x_{t},v_{m})=true\\ bright(I_{t},D_{t},<{\overset{ˇ}{I}}_{m},{\hat{I}}_{m},{\overset{ˇ}{D}}_{m},{\hat{D}}_{m}>)=true\\ depth(D_{t},<{\overset{ˇ}{D}}_{m},{\hat{D}}_{m}>)=true \end{array}\right.$$

**Figure 5:**
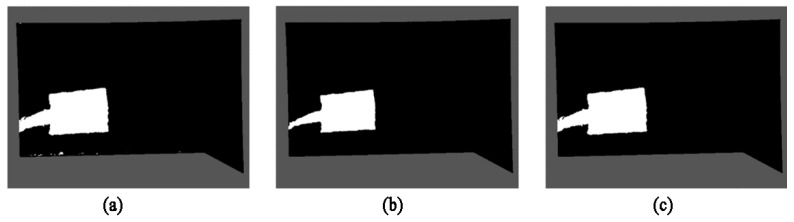
The detection result of the algorithm proposed in this paper: (**a**) Initial image of algorithm detection; (**b**) Morphological erosion image; (**c**) Morphological reconstruction image.

It can be seen from the detection results of Figure 4 that there are some noise points in the original codebook algorithm in addition to the detected foreground objects. Therefore, this paper uses morphological methods to remove the redundant noise points. Firstly, the erosion operation in the basic morphological operation of the binary image is used to remove the generated noise points. The erosion operation ablates the boundaries of objects according to the size of the structural elements, so that objects smaller than the structural elements disappear, and therefore, all noise points that do not fully contain structural elements can be removed. However, the disadvantage of erosion operation is that it will also change the shape of the foreground object to a certain extent. As shown in Figure 5b after erosion, although the sporadic noise spots are completely eliminated, the detected hand part is also eroded and thinned. Therefore, the morphological reconstruction is used to restore the image. The reconstruction involves two images, one of which is used as a marker and the other as a mask. The reconstruction method can accurately recover the image before the target area is eroded and achieve the purpose of removing the noise without changing the detection result of the foreground object. The results are shown in Figure 5c. In this paper, the morphological erosion and morphological reconstruction functions of MATLAB are used.

## 4. Results and Discussion

### 4.1. Depth Map Super-Resolution Reconstruction Based on Color Edge Guidance

In order to evaluate the effectiveness of the proposed network model framework for super-resolution reconstruction of depth maps based on color edge guidance, this paper first used the New Tsukuba Dataset [37] to train this network and then used the trained network to reconstruct the images in the Middlebury 2005 dataset [38] for qualitative and quantitative analysis. The New Tsukuba dataset is a one-minute video sequence with a frame sequence resolution of 640 × 480, including 1800 stereo matching pairs. It contains a color map, real disparity map, occlusion map and discontinuous map. Since this dataset is a scene generated by complete computer graphics (CG), the position of each coordinate can be determined, so a 100% correct depth map can be obtained. The Middlebury dataset is one of the datasets widely used in the field of depth map super-resolution reconstruction. In addition, this paper also made a comparative analysis with several other depth map super-resolution reconstruction methods, including the traditional bicubic, bilinear interpolation, reconstruction method based on second-order TGV [16], method based on nonlocal mean filtering [39] and SRCNN [5].

#### 4.1.1. Experimental Setup

The purpose of training the network is to make the network gradually learn the mapping relationship between low-resolution and high-resolution depth maps, namely, learning parameter $\theta =\{W_{i},b_{i},a_{k}\},i=1,2,3;k=1,2,3$. The data set used for training was denoted as $\left\{D_{Li},I_{bwi},D_{Hi}\right\}$, and 32×32 sub-images were cut by sliding step 24 on the low-resolution depth map $D_{L}$ after bicubic up-sampling and the optimized edge map $I_{bw}$, respectively. Since the image was not filled, the final output image size of the CNN was 20×20, so the 20×20 sub-images were intercepted at the corresponding position of $D_{H}$ as the label value for comparison in the loss function. In this paper, the mean square error (MSE) function was used as the loss function of the network, and its expression is as follows:

(17) $$L\left(\theta \right)=\frac{1}{n}\sum_{i=1}^{n}||F\left(D_{Li},I_{bwi};\theta \right)-D_{Hi}{\left|\right|}^{2}$$

where *n* represents the number of training sample sets. The use of MSE as a loss function increases the signal-to-noise ratio (PSNR) of the output, which is a commonly used objective evaluation metric for image quality. The loss function is minimized by combining the stochastic gradient descent method and the error backpropagation. The update of the weight coefficient can be expressed as:

(18) $$\left\{\begin{array}{c} \Delta_{i+1}=0.9\Delta_{i}-\eta \frac{\partial L}{\partial W_{i}^{l}}\\ W_{i+1}^{l}=W_{i}^{l}+\Delta_{i+1} \end{array}\right.$$

where l∈{1,2,3,4} represents the *l*th bias coefficient, *i* represents the number of iterations, and η is the learning rate. The network parameter settings were as follows: c=1, $n_{1}=n_{2}=64$, $n_{3}=64$, $f_{1}=f_{2}=9$, $f_{4}=5$, the learning rate of the first three convolution layers was 0.001, and the learning rate of the fourth convolution layer was 0.0001. The weight was randomly initialized by Gaussian distribution with expectation of 0 and variance of 0.01, and the biases are all initialized to 0.

In this paper, the CNN was designed and implemented based on Caffe and MATLAB. In total, 111,150 sets of sub-images obtained from 225 sets of images in New Tsukuba Dataset were selected as the training set, and 37,050 sets of sub-images obtained from 75 sets of images were selected as the test set. The low-resolution images used for training and testing were replaced by the sub-images by double-cubic down-sampling according to two and four times. The CPU of the training platform was Intel Core i5-4200H, and the GPU was Nvidia GeForce GTX 950M, 8GB memory.

#### 4.1.2. Qualitative Analysis

**Figure 6:**
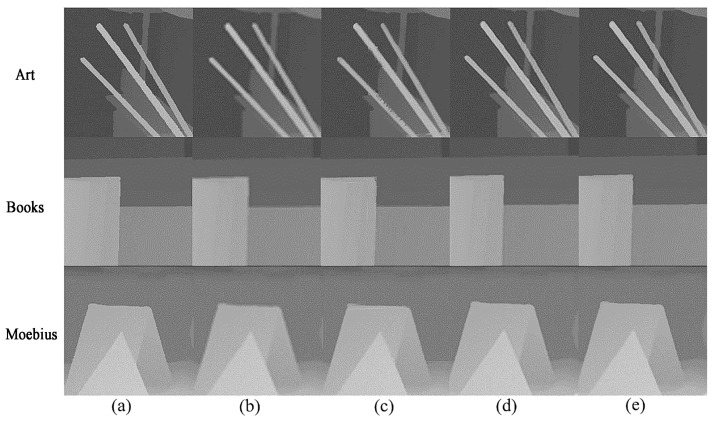
Comparison of the results of up-sampling depth maps with several methods: (**a**) Ground Truth; (**b**) Bicubic; (**c**) TVG; (**d**) SRCNN; (**e**) Method proposed in this article.

This section provides a qualitative evaluation of the experimental results. Art, Books and Moebius in the data set compare the specific details of the four times up-sampling by the above different methods, as shown in Figure 6. It can be seen from the figure that the algorithm proposed in this paper has achieved the best results in the comparison of several methods. Compared with the traditional bicubic interpolation method or the reconstruction method based on second-order TGV, the obtained image is more clear and can retain more fine structural information in the image without blurring or introducing noise. Compared with the single-image SRCNN reconstruction method, RGB edge information is added to the training network as auxiliary information, which makes the reconstructed image edge more sharp and smooth.

#### 4.1.3. Quantitative Analysis

In order to quantitatively analyze the experiment in this paper, the super-resolution reconstruction of six images in the Middlebury 2005 dataset was carried out by using the proposed algorithm and other comparison algorithms when the up-sampling coefficients were two and four in this section. The root mean square error (RMSE) was used as the evaluation index, and its expression is as follows:

(19) $$\mathrm{RMSE}=\sqrt{\frac{1}{n}\sum_{i=1}^{n}{\left(y_{i}-{\hat{y}}_{i}\right)}^{2}}$$

where $y_{i}$ represents the pixel value (the depth value) on the depth ground truth map, ${\hat{y}}_{i}$ represents the pixel value on the result map obtained by the algorithm after sampling the low-resolution depth map, and *n* represents the total number of pixels in the depth image. The RMSE index can well reflect the difference between the image reconstruction results and the real results.

Table 1 and Table 2 show the experimental results when the samples were up-sampling to two and four. Park’s results were from Reference [40], while other results were obtained by using the source code provided by the authors. The best reconstruction results in the table have been boldly displayed. It can be seen from the table that the proposed algorithm obtains the best results in the up-sampling of two and four times of the depth map. On the one hand, compared with the traditional machine learning method, the convolution-based neural network can achieve better results even than SRCNN or the shallow neural network proposed in this paper, mainly because the convolution layer obtains abundant features, which plays an important role in depth map reconstruction. On the other hand, compared with the SRCNN method based on single image reconstruction, the proposed method adds another input, namely, the color image edge information of the same scene, which is conducive to maintaining the sharpness and smoothness of the edge in the reconstructed image and achieves better experimental results.

### 4.2. Codebook Moving Target Detection Algorithm Based on RGB-D

In this paper, the SBM-RGBD dataset [41] was used to evaluate and compare the background modeling methods of moving object detection scenes in RGBD videos. The dataset provides a set of diversified true color and depth sequences obtained by Microsoft Kinect. It consists of 33 videos (about 15,000 frames), which represent typical indoor visual data captured in video surveillance and intelligent environment scenes. The resolution of the video is 640 × 480, and the depth map is 16 or 8 bits. The length of the video varies from 70 to 1400 frames. In order to verify the effectiveness of the proposed algorithm in this paper, we compare it with the original codebook algorithm in the following three scenarios set up: (1) the target is close to the background; (2) the target is similar to the background color; (3) illumination changes. Three RGB-D based moving object detection methods proposed in the last 5 years are also selected for comparison in these three scenarios, which are SRPCA [42], CwisarDH^+^ [43], and BSABU [44]. The results of these three methods are directly obtained from the results submitted by their authors on the SBM-RGBD dataset website. The experiment was divided into qualitative and quantitative analysis. The algorithm framework was built on MATLAB, and the CPU of the operating environment was Intel Core i5-4200H, 8G memory. The experimental parameters were set as shown in Table 3.

The evaluation indexes used in quantitative analysis were accuracy *P*, recall *R* and index *F*. The specific formulas are as follows:

(20) $$\left\{\begin{array}{c} P=\frac{TP}{TP+FP}\\ R=\frac{TP}{TP+FN}\\ F=2\times \frac{P\times R}{P+R} \end{array}\right.$$

where TP (True Positive) represents the number of pixels that are correctly detected as moving objects, FP (False Positive) represents the number of pixels that are falsely detected as moving objects, and FN (False Negative) represents the number of pixels that are falsely detected as background. Index *F* is the combination of *P* and *R*, and the larger the value, the better the performance of the algorithm.

#### 4.2.1. Qualitative Analysis

- Experimental Scenario One: the target is close to the background

**Figure 7:**
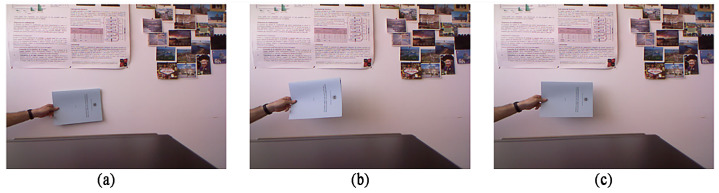
Depth sampling map of Experimental Scenario One: (**a**) the 74th frame; (**b**) the 94th frame; (**c**) the 134th frame.

**Figure 8:**
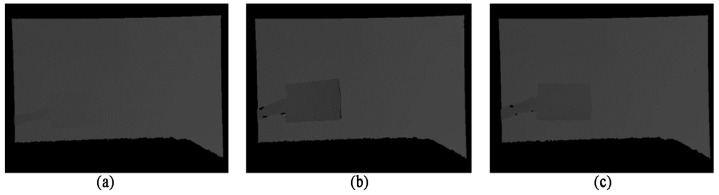
Color sampling map of Experimental Scenario One: (**a**) the 74th frame; (**b**) the 94th frame; (**c**) the 134th frame.

The content of the test frame sequence “Wall” in the data set is that the book was gradually close to and away from the lens by hand. In addition, due to the shooting light, the wall covered by the book had a large number of shadows. In this section, we selected the 74th frame, which was the nearest to the wall, the 94th frame, which was the nearest to the shot, and the 134th frame, which was the deepest shadow in the middle of the frame sequence. The color images are shown in Figure 7, and the corresponding depth images of these three frames are shown in Figure 8. From the depth map, it can be seen that the area of depth change in the scene corresponds to the hand and the book that should be detected, which also shows that the depth information is of great value in detecting objects to some extent.

**Figure 9:**
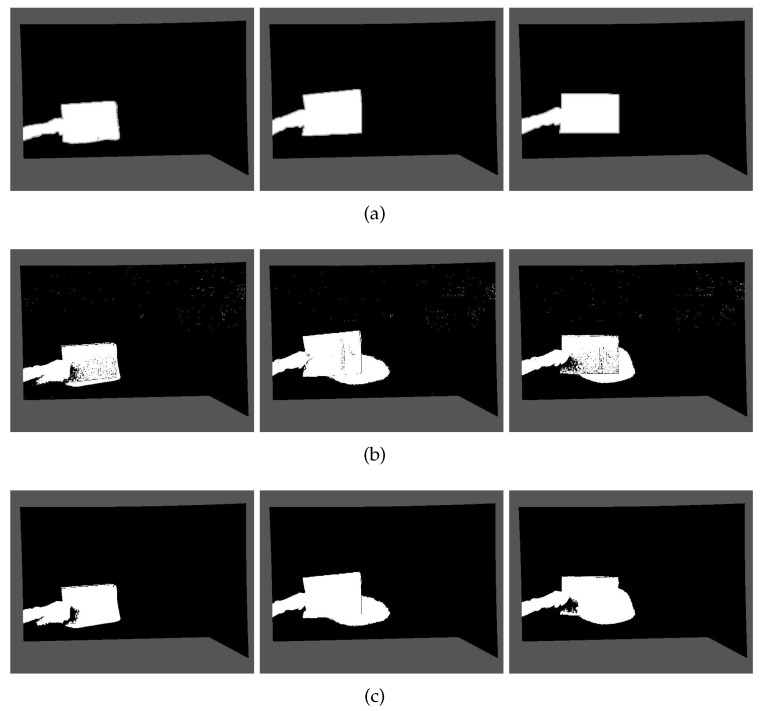

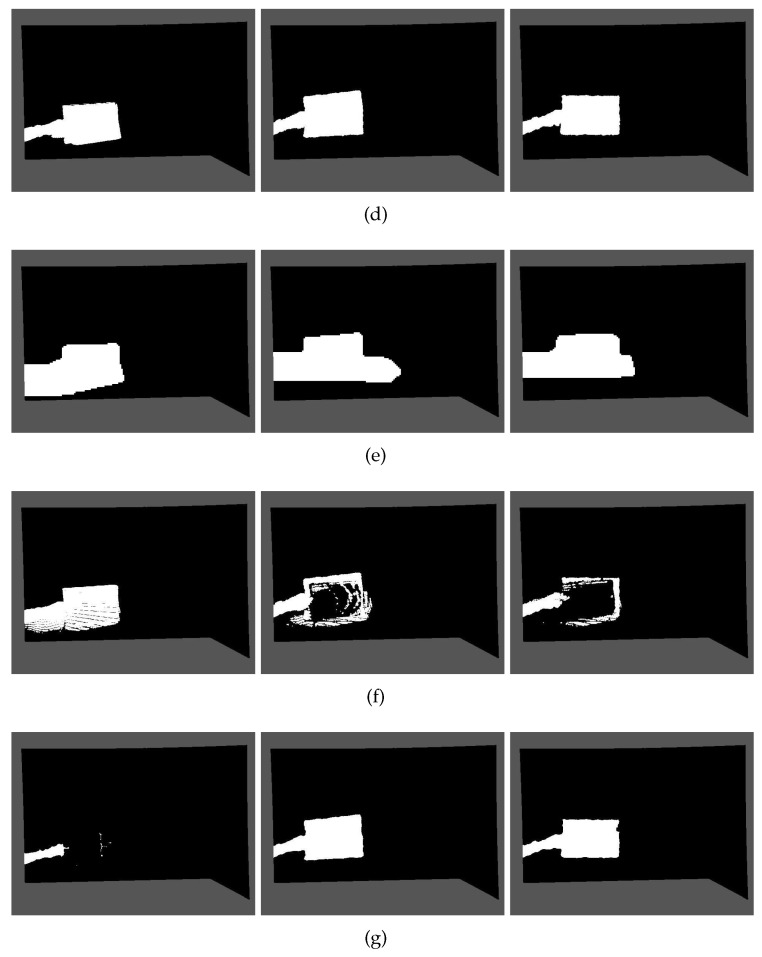
Comparison of the detection results of the two algorithms in Experimental Scenario One: (**a**) Ground truth; (**b**) The detection result of the original codebook algorithm; (**c**) The detection result of the original codebook algorithm with noise points removed using morphological operations; (**d**) The detection result of the algorithm proposed in this article; (**e**) The detection result of the SRPCA method; (**f**) The detection result of the CwisarDH^+^ method; (**g**) The detection result of the BSABU method.

The detection results are shown in Figure 9. It can be seen from the results that when the target is close to the background, the original codebook algorithm is prone to missed detection, and the detection of the hand is also prone to false detection. When the shadow of the object is deep, due to the obvious difference in light intensity and brightness between the appearance of the shadow and the background, the original algorithm is easy to misjudge the shadow as the foreground, leading to the adhesion between the moving object and the shadow. In addition, there are also some noises in the figure, and the subjective effect is poor. The detection results of the SRPCA method do not have many noise points, but some background areas are detected as the object, and the contours differ significantly from the ground truth. The contours of the CwisarDH^+^ method detection results are close to the truth, but large hole areas appear inside the object. The BSABU method has excellent detection results in the 94th and 134th frames, but when the object is closest to the background in the 74th frame, the object is almost not detected. The detection segmentation effect of the proposed algorithm is clear and free of holes and noise, which is significantly better than the original algorithm and the other three methods.

- Experimental Scenario Two: the target is similar to the background color

**Figure 10:**
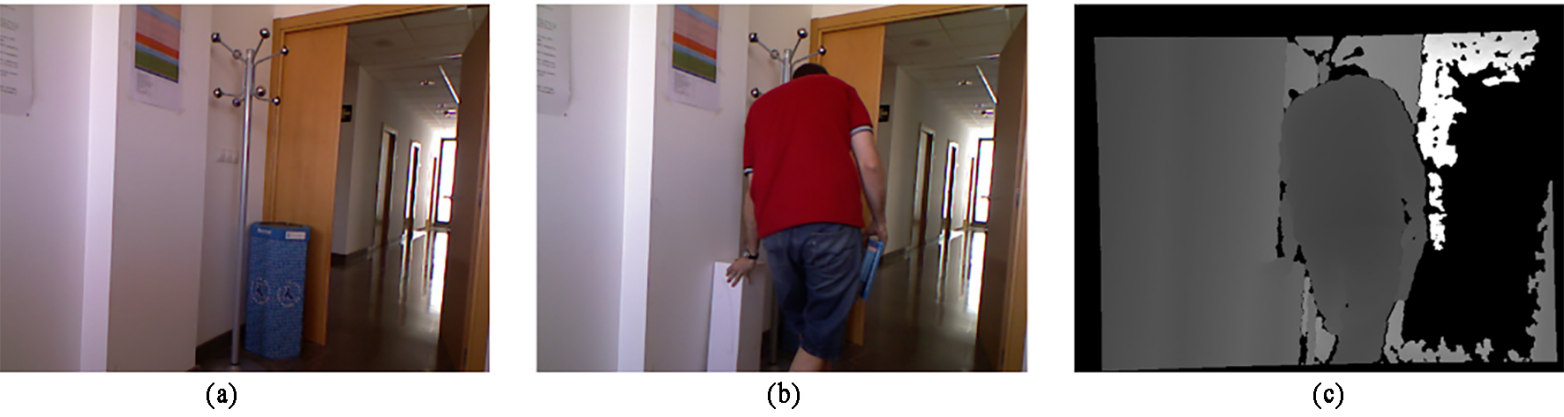
Sampling map of Experimental Scenario Two: (**a**) Background image; (**b**) Color image of the 258th frame; (**c**) Depth image of the 258th frame.

**Figure 11:**
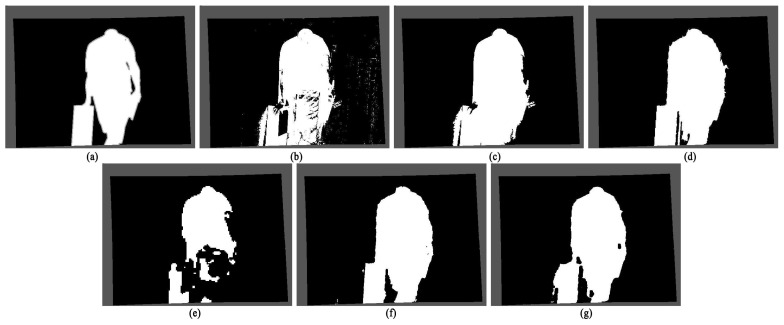
Comparison of the detection results of the two algorithms in Experimental Scenario Two: (**a**) Ground truth; (**b**) The detection result of the original codebook algorithm; (**c**) The detection result of the original codebook algorithm with noise points removed using morphological operations; (**d**) The detection result of the algorithm proposed in this article; (**e**) The detection result of the SRPCA method; (**f**) The detection result of the CwisarDH^+^ method; (**g**) The detection result of the BSABU method.

According to experience, when the moving target is similar to the background color, it is easy to lead to missed or false detection of target pixels. Therefore, this paper selected the “Hallway” frame sequence in the data set to compare the effectiveness of the two algorithms. The man in the video walked with the same box as the white wall color, and the pants color was similar to the barrel color. In the experiment, the 258th frame was selected. The sampling images of the experiment are shown in Figure 10, and the detection results are shown in Figure 11.

It can be seen from the test results that due to the similar color between the wall and the box, and between the pants and the bucket, the original algorithm has many false detections and objects are not coherent. In addition to some details such as the gap between the arms, the algorithm proposed in this paper maintains a relatively complete shape of the box and the person. The detection result of the SRPCA method is incomplete in the legs of the characters with more holes, while the detection results of both the CwisarDH^+^ method and the BSABU method in this scenario are closer to the ground truth, and the gaps between the person and the box can be detected better. The result of BSABU is slightly better than the algorithm proposed in this paper, which is the best result among these methods.

- Experimental Scenario Three: sudden illumination changes

**Figure 12:**
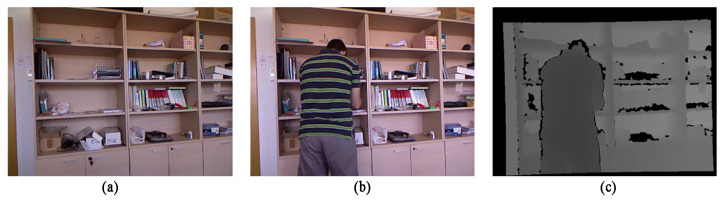
Sampling map of Experimental Scenario Three: (**a**) Background image; (**b**) Color image of the 390th frame; (**c**) Depth image of the 390th frame.

**Figure 13:**
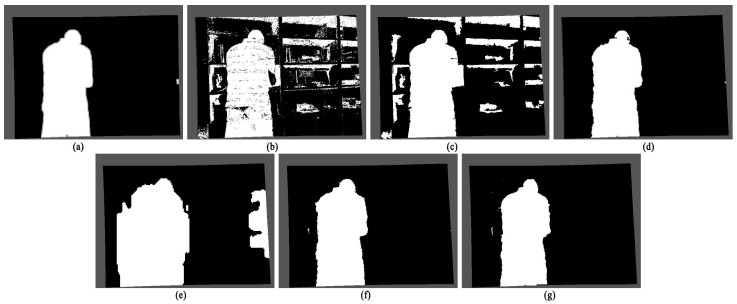
Comparison of the detection results of the two algorithms in Experimental Scenario Three: (**a**) Ground truth; (**b**) The detection result of the original codebook algorithm; (**c**) The detection result of the original codebook algorithm with noise points removed using morphological operations; (**d**) The detection result of the algorithm proposed in this article; (**e**) The detection result of the SRPCA method; (**f**) The detection result of the CwisarDH^+^ method; (**g**) The detection result of the BSABU method.

When using the background modeling algorithm, it often occurs after the background model is established in the training process, and the illumination conditions of the test frame change, resulting in many false detections of the test results. In this paper, we select the “Shelves” frame sequence from the test set, in which a man enters a room and turns on the light, causing a sudden change in illumination. In the experiment, the 390th frame was selected. The sampling images of the experiment are shown in Figure 12, and the truth map and the detection results of the two algorithms are shown in Figure 13.

It can be seen from the test results that due to the difference between the illumination brightness in the detection and the background modeling, in the original algorithm, a large number of pixels are mistaken for the foreground due to the brightness change. The algorithm proposed in this paper is able to detect the foreground more completely due to the addition of depth information as an auxiliary judgment, and the detection is greatly improved because the depth is not affected by the change of illumination. The detection result of the SRPCA method is much different from the ground truth, and there are more false detections. Although the detection results of both the CwisarDH^+^ and the BSABU methods are closer to the ground truth and can clearly and completely detect the human form, the detection results have spikes on the edges and generate a little noise on the left side of the figure compared to the algorithm proposed in this paper. Therefore, the algorithm in this paper subjectively achieves the best results in this scenario.

#### 4.2.2. Quantitative Analysis

Table 4 lists the value of the quantitative index *F* used to evaluate the detection effect of the several methods in the above three scenarios, and the best result for each experiment has been shown in bold. From the data in the table, it can be seen that the algorithm proposed in this paper achieves the best results in both the “Wall” and the “Shelves” experiments, and its F-value is only 2.27% lower than the best result in the “Hallway” experiment, although it does not achieve the best result. Although the result of the CwisarDH^+^ and the BSABU methods in the “Hallway” experiment is slightly better than the proposed algorithm, the result of the CwisarDH^+^ method in the “Wall” experiment at the 74th frame is only 0.2736, which is much lower than the 0.9916 of the proposed algorithm, and the result of the BSABU method in the “Wall” experiment at the 94th and 134th frames is only 0.5737 and 0.4932, which is much lower than the 0.9953 and 0.9889 of the proposed algorithm. Therefore, combining all the experimental results, the algorithm proposed in this paper achieves the best result among all the methods compared, and it has significantly improved compared with the original algorithm, and the detection results in the experiments of each scenario are excellent.

The codebook algorithm requires only a small amount of memory to save the model and has a small amount of computation, so it can run for a long time and the detection speed is also fast. In this paper, the original codebook algorithm is improved by adding depth information, and the stability of depth information largely improves the defect that the original algorithm has difficulty in accurately detecting foreground targets when shadow and illumination changes affect it. Finally, the detection experiments were carried out using the proposed algorithm in three scenes: dark shadows, similar color of objects and background, and illumination changes. The detection results are compared with the other three RGB-D based methods, which further qualitatively and quantitatively verify the effectiveness of the improved algorithm.

## 5. Conclusions

In this paper, we propose a color edge-guided depth map super-resolution convolutional neural network framework and RGB-D-based codebook moving object detection algorithm. Our work improves the super-resolution reconstruction and moving object detection, and the validity of the model framework and algorithm is verified on different datasets. Firstly, according to the corresponding relationship between the color map and the depth map, the low-resolution depth map and the high-resolution optimized color edge map of the same scene are simultaneously input to the network, with the aim of using the color edge map to guide the reconstruction of the depth map. The reconstructed results are then compared with several traditional super-resolution algorithms, and the results show that the proposed model framework effectively improves the quality of the depth map after super-resolution. Secondly, we add the depth information of the scene as an auxiliary dimension based on the original codebook algorithm to better segment moving objects by using the property that depth information is almost immune to shadows, light changes, and color interference. Finally, the post-processing of morphological erosion and morphological reconstruction is used to reduce noise and optimize the detection results. We compare the proposed algorithm with three other recent RGB-D based methods, and the results show that the proposed algorithm significantly improves the effectiveness of the original algorithm in detecting objects in three scenarios, and it achieves the best results overall among all methods, proving the effectiveness of the proposed method in this paper.

In future research, we consider adding deconvolution layers to replace the up-sampling in pre-processing, increasing the number of network layers, increasing the nonlinearity, and improving the network fitting ability to make the reconstructed accuracy higher. In addition, from the aspect of moving object detection, the proposed algorithm sets the threshold, boundary constant and other hyperparameters to a fixed value, which is difficult to guarantee good detection results in all scenarios, and it may cause problems such as large amounts of noise and holes in detection. Therefore, the subsequent research considers changing these constants to adaptive to expand the applicability of the algorithm in different scenes.

## Figures and Tables

**Table 1:** Comparison of RMSE results of several methods (Art/Books/Moebius).

	Art		Books		Moebius	
	**2×**	**4×**	**2×**	**4×**	**2×**	**4×**
Bicubic	2.630	3.872	1.045	1.604	0.870	1.329
Bilinear	2.875	4.150	1.132	1.698	0.955	1.431
Park et al.	2.833	3.498	1.088	1.530	1.064	1.349
Ferstl et al.	3.164	3.761	1.370	1.770	1.153	1.497
SRCNN	1.924	2.651	0.793	1.150	0.726	1.049
The proposed model	**1.051**	**2.222**	**0.437**	**0.873**	**0.505**	**0.905**

**Table 2:** Comparison of RMSE results of several methods (Dolls/Laundry/Reindeer).

	Dolls		Laundry		Reindeer	
	**2×**	**4×**	**2×**	**4×**	**2×**	**4×**
Bicubic	0.910	1.309	1.601	2.396	1.928	2.814
Bilinear	0.987	1.400	1.738	2.548	2.096	3.014
Park et al.	0.963	1.301	1.552	2.132	1.834	2.407
Ferstl et al.	1.164	1.435	1.891	2.681	2.546	3.165
SRCNN	0.8	1.124	1.095	1.742	1.378	2.013
The proposed model	**0.679**	**1.063**	**0.663**	**1.321**	**0.803**	**1.687**

**Table 3:** Experimental parameters settings of moving target detection algorithm.

Parameters	Values	Parameters	Values	Parameters	Values
α	0.75	β	1.3	$\varepsilon_{1}$	5
$\alpha_{d}$	0.97	$\beta_{d}$	1.05	$\varepsilon_{t}$	25
$\alpha_{2}$	0.45	$\beta_{2}$	1.4	*k*	8

**Table 4:** Comparison of the F-values of the proposed method with several RGB-D based methods in recent years for 3 scenarios. The results with the best performance in each scenario have been bolded.

	Wall			Hallway	Shelves
	74th Frame	94th Frame	134th Frame	258th Frame	390th Frame
SRPCA [42]	0.8192	0.7846	0.8433	0.7835	0.7598
CwisarDH^+^ [43]	0.8966	0.5737	0.4932	0.9736	0.9945
BSABU [44]	0.2736	0.9892	0.9770	**0.9843**	0.9912
Original algorithm	0.9182	0.8642	0.8367	0.8756	0.7102
Original algorithm after morphological operations	0.9374	0.8704	0.8636	0.9199	0.7748
Proposed algorithm	**0.9916**	**0.9953**	**0.9889**	0.9620	**0.9960**

## Data Availability

CDNet2014, New Tsukuba, Middlebury 2005, SBM-RGBD datasets are available in the public domain.
